# Transient Multivalent Nanobody Targeting to CD206-Expressing Cells via PH-Degradable Nanogels

**DOI:** 10.3390/cells9102222

**Published:** 2020-10-01

**Authors:** Maximilian Scherger, Evangelia Bolli, Ana Rita Pombo Antunes, Sana Arnouk, Judith Stickdorn, Alexandra Van Driessche, Hansjörg Schild, Stephan Grabbe, Bruno G. De Geest, Jo A. Van Ginderachter, Lutz Nuhn

**Affiliations:** 1 Max Planck Institute for Polymer Research, Ackermannweg 10, 55128 Mainz, Germany; maximilian.scherger@mpip-mainz.mpg.de (M.S.); judith.stickdorn@mpip-mainz.mpg.de (J.S.); 2 Myeloid Cell Immunology Lab, VIB Center for Inflammation Research, Pleinlaan 2, 1050 Brussels, Belgium; evangeliabolli@gmail.com (E.B.); Ana.Rita.Pombo.Antunes@vub.be (A.R.P.A.); Sana.Arnouk@vub.be (S.A.); 3 Lab of Cellular and Molecular Immunology, Vrije Universiteit Brussel, Pleinlaan 2, 1050 Brussels, Belgium; 4 Department of Pharmaceutics, Ghent University, Ottergemsesteenweg 460, 9000 Ghent, Belgium; alexandra.vandriessche@ugent.be (A.V.D.); br.degeest@ugent.be (B.G.D.G.); 5 Institute of Immunology, University Medical Center of Johannes Gutenberg-University Mainz, Obere Zahlbacher Straße 63, 55131 Mainz, Germany; schild@uni-mainz.de; 6 Department of Dermatology, University Medical Center of Johannes Gutenberg-University Mainz, Obere Zahlbacher Straße 63, 55131 Mainz, Germany; stephan.grabbe@unimedizin-mainz.de

**Keywords:** nanogel, nanobody, targeting, RAFT polymerization, click chemistry, CD206, TAM, M2 macrophage, multivalency, targeting

## Abstract

To target nanomedicines to specific cells, especially of the immune system, nanobodies can be considered as an attractive tool, as they lack the Fc part as compared to traditional antibodies and, thus, prevent unfavorable Fc-receptor mediated mistargeting. For that purpose, we have site-specifically conjugated CD206/MMR-targeting nanobodies to three types of dye-labeled nanogel derivatives: non-degradable nanogels, acid-degradable nanogels (with ketal crosslinks), and single polymer chains (also obtained after nanogel degradation). All of them can be obtained from the same reactive ester precursor block copolymer. After incubation with naïve or MMR-expressing Chinese hamster ovary (CHO) cells, a nanobody mediated targeting and uptake could be confirmed for the nanobody-modified nanocarriers. Thereby, the intact nanogels that display nanobodies on their surface in a multivalent way showed a much stronger binding and uptake compared to the soluble polymers. Based on their acidic pH-responsive degradation potential, ketal crosslinked nanogels are capable of mediating a transient targeting that gets diminished upon unfolding into single polymer chains after endosomal acidification. Such control over particle integrity and targeting performance can be considered as highly attractive for safe and controllable immunodrug delivery purposes.

## 1. Introduction

Nano-sized carriers can decouple drugs from their pharmacokinetic profile and facilitate their delivery to desired sites of actions [1]. Thus far, this has been intensely investigated for classical anticancer chemotherapeutics [2,3], primarily inspired by passive accumulation of circulating nanoparticles into highly vascularized tumors through the so-called enhanced permeability and retention (EPR) effect [4,5,6]. In parallel, strategies of actively targeting tumors have been discussed [7], mostly motivated, e.g., by the Ringsdorf model of polymer therapeutics [8,9,10]. A targeting ligand is considered to guide the macromolecular drug carrier to the receptor, affording cell-specific drug delivery. Throughout the years this principal has mostly been realized by direct coupling of drugs onto antibodies yielding so-called antibody-drug conjugates (ADCs) [11]. However, due to limited conjugation efficiencies and unwanted protein aggregation, drug encapsulation strategies into nanocarrier followed by surface decoration with antibodies still seem favorable [12]. Nevertheless, one has to take into account that clustering of antibody’s Fc-part by the nanocarrier can still mediate non-specific mis-targeting to Fc-receptor bearing cells, mostly of the reticuloendothelial system, thus, losing the desired targeting specificity [13].

Alternatively, single chain antigen binding fragments, so-called nanobodies, derived from camelid heavy chain-only antibodies [14,15], seem highly attractive to promote better active nanocarrier targeting. With their molecular weight of about 15 kDa, they are 10 times smaller than conventional antibodies and lack the Fc-part. Moreover, in contrast to enzymatically derived antibody fragments (Fabs), they consist of a single protein chain which can be produced recombinantly on a large scale and modified for site-selective chemical modifications [16]. The latter can be used to introduce chemoselective linkers instead of non-specific conjugation sites [17,18] that could potentially interfere with the target binding site [19,20,21,22,23].

Among several targets addressable by nanobodies, the Macrophage Mannose Receptor (MMR, CD206) is expressed on tumor-associated macrophages (TAM) that usually govern the immunosuppressive state of the tumor microenvironment [24,25]. Such MMR expressing macrophages are considered as highly attractive for immunodrug delivering nanomedicines, especially towards altering the macrophages’ phenotype and re-establishing antitumor immunity [26]. A nanobody specific for MMR has been generated and directly exploited for diagnostic and therapeutic purposes [27,28,29,30] showing its superior benefits compared to full antibodies [31,32,33]. We have recently applied this nanobody for site-specific modification and conjugation to a customizable nanogel drug carrier platform, which then promoted effective nanoparticle delivery to MMR-expressing TAMs in vitro, ex vivo, and in vivo of tumor-bearing mice [34].

The applied nanogel platform is derived from amphiphilic reactive ester block copolymers that self-assemble into reactive precursor micelles whose cores can be crosslinked and converted into double hydrophilic nanogels [35]. During this process, further functional entities can be covalently integrated into the core of the resulting particles, thereby enabling, e.g., therapeutic oligonucleotide [36] or immunodrug delivery [37]. Interestingly, by applying heterotelechelic precursor block copolymers, orthogonal reactive groups can further be exposed onto the nanogel surface for additional nanogel derivatization [34,38,39,40,41]. In our latest study, we have used an azido-modified chain transfer reagent [42] to generate amphiphilic reactive ester block copolymers and, subsequently, azido surface decorated nanogels. They were used for copper-free strain promoted azide alkyne cycloaddition (SPAAC) ligation [43,44] of C-terminal mono-dibenzocyclooctyne (DBCO) modified anti-MMR nanobodies [34].

Besides nanogel corona modification, their cores also allow stimuli-responsive modification with pH-degradable cross-linkers that control the integrity of the carrier over time. By crosslinking these nanogels with an acid-degradable ketal crosslinker, while labeling each polymer strand with a FRET responding fluorescent dye, one can even monitor particle disintegration into single fully water-soluble polymer chains upon cellular internalization and translocation into acidic lysosomal compartments [45]. Interestingly, nanogel unfolding into single polymer chains also seem to influence the performance of the co-delivered drug [39,46]. However, we have thus far not investigated how this might also affect the targeting capacity of the surface-immobilized nanobody.

To address this, we have utilized the reactive ester precursor block copolymer platform and synthesized in parallel three types of dye-labeled nanogel derivatives: non-degradable nanogels, acid-degradable nanogels (with ketal crosslinks), and single polymer chains (as their degraded version). All of them could be conjugated with DBCO-modified anti-MMR nanobodies. The carriers’ uptake into MMR-expressing Chinese hamster ovary (CHO) cells could be monitored and compared by confocal microscopy and flow cytometry. Our findings reveal superior targeting and uptake performance of the intact nanocarrier displaying nanobodies in a multivalent fashion. Interestingly, the pH-responsive degradation potential of the ketal-crosslinked nanogel only facilitates transient targeting, which gets diminished upon unfolding into single polymer chains that would be small enough to be renally cleared from the body. Altogether, such control over particle integrity and targeting performance seems highly suitable for safe and controllable immunodrug delivery purposes.

## 2. Materials and Methods

### 2.1. Materials

Unless otherwise stated, chemicals were all obtained from Sigma-Aldrich (Taufkirchen, Germany) and Acros Organics (Geel, Belgium) and used as such. Oregon Green cadaverine was purchased from Thermo Fisher (Dreieich, Germany) and dialyses were conducted in Spectra/Por3 membranes obtained from Spectrum Labs (New Brunswick, NJ, USA) with a molecular weight cut-off of 1000 g/mol.

For nanogel synthesis and subsequent conjugation with MMR-targeting nanobodies, all required building blocks including the reactive precursor polymer N_3_-P(MEO_3_MA)_12_-*b*-P(PFPMA)_40_ and the mono-DBCO-PEG_4_-maleimide functionalized anti-MMR nanobody were generated as described in our earlier work [34].

### 2.2. Instrumentation

UV-vis spectra were recorded by a Shimadzu UV-1650PC spectrophotometer (Duisburg, Germany) inside 1 cm × 1 cm quartz cells.

Dynamic light scattering measurements (DLS) were recorded by a Zetasizer Nano S (Malvern Instruments Ltd., Malvern, U.K.) equipped with a HeNe laser (λ = 633 nm) and a detector at 173° scattering angle. All data were analyzed by cumulant fitting for z-average mean diameter and polydispersity index (PDI), while the constrained regularization method for inverting data represented by linear or integral equations (CONTIN fitting method) was applied for particle diameter size distribution.

Sodium dodecyl sulfate poly(acrylamide) gel electrophoresis (SDS-PAGE) was run at 180 V for 45 min on commercial 4–20% polyacrylamide gradient gels (Mini-PROTE0AN TGX gels) with a Mini-PROTEAN Tetra Cell from Bio-Rad (Feldkirchen, Germany). Two µg of the respective nanobody samples were diluted in phosphate-buffered saline PBS and then mixed with 4× Laemmli sample buffer at a ratio of 3:1 (*v/v*) before they were loaded onto the SDS-PAGE gels. Protein bands could be stained afterwards by Coomassie Blue and then recorded by a BioRad Gel Doc system using the corresponding BioRad Gel Doc software to determine protein conjugation efficiencies by densitometry.

Flow cytometry analyses (FACS) were performed with a BD Accuri C6 (BD Biosciences, Erembodegem, Belgium) and the obtained data processed by FlowJo software package.

Confocal microscopy images were recorded on a Leica DMI6000B scope attached to an Andor DSD2 scanner and equipped with a 1.40 NA 63 × oil immersion objective. Images were processed by ImageJ software package.

### 2.3. Synthesis of (Non-)Degradable Nanogels and Non-Crosslinked, Soluble Polymers

As previously reported [34], the reactive precursor block copolymer N_3_-P(MEO_3_MA)_12_-*b*-P(PFPMA)_40_ was kept under nitrogen atmosphere in anhydrous DMSO at 10 mg/mL and could be dispersed into block copolymer micelles by sonication. For fluorescent labeling, 2.0 mL of this micellar N_3_-P(MEO_3_MA)_12_-*b*-P(PFPMA)_40_ solution (20.0 mg; 69.2 µmol PFP-ester; 1.0 eq.) was first treated with Oregon Green cadaverine (65.5 µL of a 2.5 mg/mL stock solution in DMSO, 0.35 µmol, 0.005 eq.) and triethylamine (TEA) (2.4 µL; 17.3 µmol; 0.25 eq.) overnight at room temperature. For non-degradable crosslinking, 2,2′-(ethylenedioxy)bis(ethylamine) (5.1 µL; 34.6 µmol; 0.5 eq.) and TEA (28.9 µL; 207.6 µmol; 3.0 eq) were then added and the reaction mixture kept stirring overnight at 50 °C. In case of pH-degradable crosslinking, 2,2-bis(aminoethoxy)propane (5.5 µL; 34.6 µmol; 0.5 eq.) and TEA (28.9 µL; 207.6 µmol; 3.0 eq) were added, while for fabricating non-crosslinked, soluble polymers, 2-aminoethanol (12.4 µL; 207.6 µmol; 3.0 eq.) was added with TEA (28.9 µL; 207.6 µmol; 3.0 eq). After stirring overnight at 50 °C, additional 2-aminoethanol (12.4 µL; 207.6 µmol; 3.0 eq.) and TEA (28.9 µL; 207.6 µmol; 3.0 eq) was added to all samples and stirred for another day at 50 °C in order to guarantee complete removal of probable remaining pentafluorophenyl esters. Afterwards, all reaction mixtures were transferred into dialysis membranes and dialyzed against water (supplemented with 0.015 M ammonia, to prevent premature ketal hydrolysis) for five days. Subsequent lyophilization afforded Oregon Green labeled (non-)degradable nanogels or non-crosslinked polymers as voluminous dry and orange powder (average yield 85%). It could be readily re-dispersed in PBS at given concentrations supported by sonication and used as such prior to each experiment.

### 2.4. Nanobody Conjugation to (Non-)Degradable Nanogels and Non-Crosslinked, Soluble Polymers

As earlier reported [34], anti-MMR nanobodies with one single DBCO-PEG_4_-maleimide attached to a cysteine located at their C-terminus could be conjugated to the azides exposed on the surfaces of the (non-)degradable nanogels or non-crosslinked single polymer chains via strain promoted alkyne azide cycloaddition (SPAAC). As an example, 100 µL of a 10 mg/mL nanogel or polymer sample in PBS (with 37.1 nmol accessible azides, as determined previously [34], corresponding to 30.0 eq.) were mixed with 97.7 µL of 0.2 mg/mL DBCO-nanobody solution in PBS (1.24 nmol, 1.0 eq.) and incubated at room temperature for 16 h. Note that corresponding samples of (non-)crosslinked nanogels or soluble polymers with equivalent amounts of PBS instead of DBCO-nanobody were prepared and used as controls, accordingly. Afterwards, an aliquot of each sample was taken for SDS-PAGE to check ligation efficiency of SPAAC-mediated nanobody conjugation. All samples were stored at 4 °C and used as such for further experiments.

### 2.5. Uptake of Anti-MMR Nanobody-Functionalized (Non-)Degradable Nanogels and Non-Crosslinked, Soluble Polymers by CHO^MMR+^ and CHO^MMR−^ Cells

#### 2.5.1. Cell Culture

Immortalized Chinese Hamster Ovary cells (obtained from ATCC, Molsheim Cedex, France) that do not express the MMR/CD206 receptor (CHO^MMR−^ cells) could be cultured in Dulbecco’s Modified Eagle Medium (DMEM) supplemented with 10% fetal bovine serum, 1% penicillin/streptomycin, 2 mM l-glutamine, and 1 mM sodium pyruvate. For genetically modified Chinese Hamster Ovary cells that stably express the MMR/CD206 receptor according to reported literature procedures [47,48,49,50,51] (CHO^MMR+^ cells), their culture medium had to be further supplemented with 0.06% geneticin (G418). Both cell lines were kept at 37 °C in a controlled, sterile environment of 95% relative humidity and 5% CO_2_.

#### 2.5.2. Flow Cytometry

For determining particle uptake by flow cytometry, CHO^MMR+/−^ cells were seeded at 200,000 cells per well in 900 μL culture medium into 24-well titer plates and left overnight for adhesion. Next, they were pulsed with 100 μL of the respective 0.5 mg/mL (non-)crosslinked nanogel or soluble polymer solution (with or without conjugated nanobody) in PBS (affording a total sample concentration of 50 μg/mL). Each sample was run in triplicate and incubation time lasted for 4 h at 37 °C. Before flow cytometric analysis, culture medium was aspirated, cells were washed with 1 mL PBS and then incubated with cell dissociation buffer (500 μL) for 15 min at 37 °C. Afterwards, detached cells could be transferred into Eppendorf tubes and centrifugation for 10 min at 350 g and 5 °C. Their supernatant was aspirated and the pellets suspended in 200 μL of PBS. All samples were kept on ice prior to flow cytometric analysis by a BD Accuri C6 (BD Biosciences). All experiments were conducted in triplicate (*n* = 3).

#### 2.5.3. Confocal Microscopy

In total, 190 µL culture medium containing 50,000 CHO^MMR+/−^ cells were transferred into Willco-Dish glass bottom dishes and incubated overnight for adhesion. Afterwards, 10 μL of a 0.5 mg/mL (non-)crosslinked nanogel or soluble polymer solution (with or without conjugated nanobody) in PBS were added affording again a total nanogel concentration of 50 μg/mL. After incubation for 4 h at 37 °C, medium was aspirated and cells were washed with PBS twice and then fixed with 200 μL of 4% paraformaldehyde (15 min at 37 °C). In parallel, a staining solution was prepared containing Hoechst (20 μL of a 1 mg/mL stock in DMSO) and Alexa Fluor 555 phalloidin (10 μL of a 1 mg/mL stock in methanol) in PBS with 1% of BSA (4.0 mL). After washing the fixated cells again for a times with PBS, 200 μL of the staining solution was added and again incubated for 30 min at 37 °C. Finally, each sample well was washed several times with PBS and immediately imaged by confocal microscopy on a Leica DMI6000 B inverted microscope equipped with an oil immersion objective (Leica, 63 ×, NA 1.40) and attached to an Andor DSD2 confocal scanner. All images were processed with the ImageJ software package.

#### 2.5.4. Statistical Analysis

Data are shown as mean values + standard deviation. To calculate statistical significance of the mean values, Student’s t-test with Welch’s correction was performed with Graph Pad Prism 8 software (*: *p* ≤ 0.01; **: *p* ≤ 0.001; ***: *p* ≤ 0.0001).

## 3. Results and Discussion

We have recently reported on the versatility of reactive precursor polymer derived nanogels in immunodrug delivery [37] and on the opportunity of using heterotelechelic precursor polymers for their click chemistry based surface modification [38]. More recently, we have shown that this is also possible via copper-free strain promoted azide alkyne cycloaddition (SPAAC) [43,44] and fabricated C-terminal mono-dibenzocyclooctyne (DBCO) modified anti-MMR nanobodies for that purpose [34].

**Figure 1:**
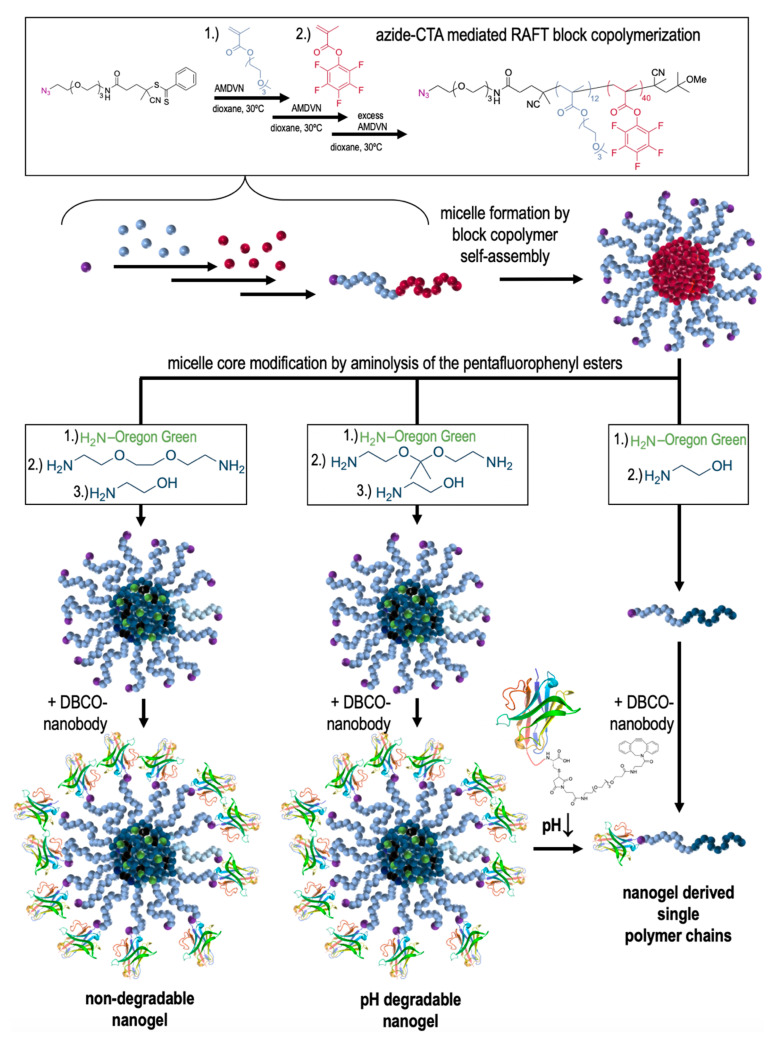
Nanobody surface-modified (non)-degradable nanogels and single polymer chains. Amphiphilic reactive precursor micelles with azido end groups can be obtained by controlled radical polymerization (RAFT) using an azide-functionalized chain transfer agent (CTA). They are first self-assembled into reactive precursor micelles and then converted into non-degradable or degradable nanogels with surface exposing azido groups. The latter can be addressed by C-terminal mono-dibenzocyclooctyne (DBCO)-modified nanobodies that react with the azido moieties and, thus, get immobilized on the nanogel surface. The ketal-crosslinked nanogels can additionally unfold into single polymer chains that are as well accessible by converting the precursor micelles with excess 2-aminoethanol. Their azido group can be fused with the nanobody, too, affording single polymer nanogel conjugates that are similarly obtained upon acidic pH-induced unfolding of the nanobody-modified, degradable nanogels.

However, our nanogel platform also allows for generating nanogels with different degradation profiles by reactive ester aminolysis with primary amines, as summarized by Figure 1. By using 2,2′-(ethylenedioxy)bis(ethylamine) as bifunctional crosslinker for the P(MEO_3_MA)-*b*-P(PFPMA) derived reactive precursor micelles, a non-degradable version is generated. When applying 2,2-bis(aminoethoxy)propane as crosslinker, a ketal-crosslinked nanogel is obtained with an acidic pH-responsive degradation profile. The nanogel unfolds into single polymer chains upon exposure to endosomal pH [45]. The resulting double hydrophilic block copolymers can also be synthetically accessed by treating the P(MEO_3_MA)-*b*-P(PFPMA) precursor micelles with excess 2-aminoethanol.

Interestingly, by using the azido-modified chain transfer reagent 1-azido-16-cyano-13-oxo-3,6,9- trioxa-12-azaheptadecan-16-yl benzodithioate [42] during the block copolymer synthesis of the reactive precursor polymers, azido functionalities are installed on the hydrophilic end of the block copolymers and consequently exposed to the surface of the resulting nanogels. They are, therefore, accessible for mono-DBCO modified nanobodies, as recently reported [34], and could, thus, be used in this study to generate nanobody-decorated pH-degradable nanogels or their degraded single polymer chains, as summarized by Figure 1.

During the fabrication process, a mono-amine functionalized dye (Oregon Green cadaverine) is further included to provide these three types of nanocarriers (degradable or non-degradable nanogels or soluble polymers) with a similar degree of fluorescent labeling per polymer.

**Figure 2:**
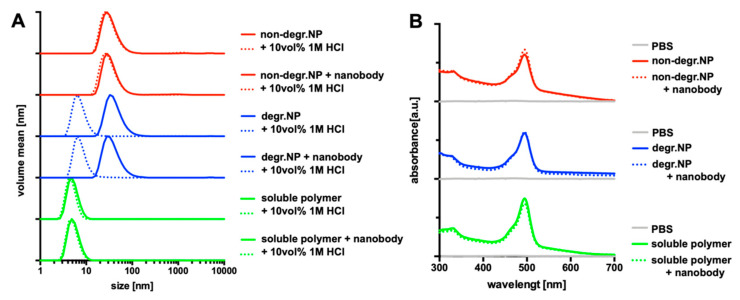
Nanobody conjugation does not affect the physicochemical properties of the (non-)degradable nanogels or soluble polymers. (**A**) DLS size distribution of the different types of nanogel and polymer samples before (continuous line) and after addition of 10 vol% 1 M HCl to the PBS sample (dashed line), affording endosomal pH conditions (pH 4–5). They lead to a disassembly only of degradable, ketal-crosslinked nanogels into soluble polymers exclusively. (**B**) UV vis spectra of the samples with (dashed line) and without (continuous line) nanobody conjugated to (non-)degradable nanogel surface or the azido end group of the soluble polymers showing similar fluorescent dye loading for each species.

After the fabrication process, all types of nanocarriers were further characterized by dynamic light scattering providing hydrodynamic diameters between 50 to 60 nm for the nanogels and below 10 nm for the soluble polymers, as shown in Figure 2A and summarized by Table 1. Moreover, only the ketal-crosslinked nanogels provided the acidic pH-responsive degradation profile (Figure 2A). Upon exposure to endosomal pH-conditions by acidification of the PBS solution with 10 *v/v*% 1 M HCl (affording an endosomal pH value of 4–5), only the ketal-crosslinked nanogels are able to unfold into single polymer chains yielding sizes in analogy to the double hydrophilic polymer chains, which were directly obtained by aminolysis of the precursor micelles with excess 2-aminoethanol (compare Figure 1).

**Figure 3:**
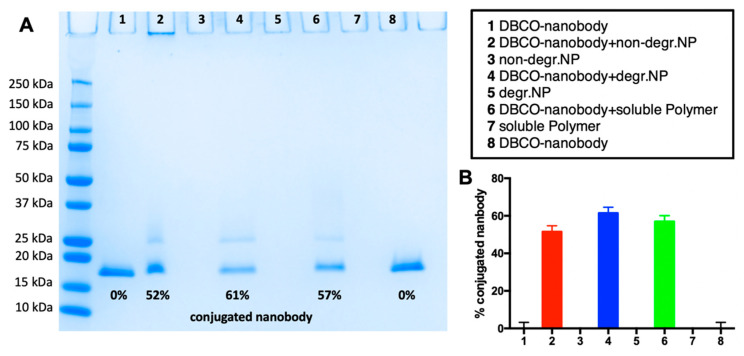
Conjugation of DBCO-bearing nanobodies to the azide surface, exposing (non-)degradable nanogels or azide end group exposing soluble polymers. (**A**) Sodium dodecyl sulfate poly(acrylamide) gel electrophoresis (SDS-PAGE) and (**B**) derived conjugation efficiency obtained by densitometry of the corresponding protein bands of the SDS-PAGE gel.

Next, DBCO-modified nanobodies were conjugated via SPAAC to the azido groups on the surface of the nanogels or the corresponding end groups of the soluble polymers, as summarized in Figure 1. Mono-DBCO modification of the MMR (CD206) targeting nanobodies could be achieved by selectively addressing their C-terminal cysteine with a DBCO-PEG_4_-maleimide, as recently reported [34]. Afterwards, both components, azido-bearing polymeric carriers and DBCO-equipped nanobodies, could be conjugated by simply mixing them in PBS and incubating them at room temperature for 16 h. We afterwards performed SDS-PAGE to verify reduction of the amount of free nanobody as a result of ligation to the nanogel. In Figure 3A the results of a corresponding conjugation for all three carrier species are summarized. In general, under the applied conditions a 50–60% conjugation efficiency could be obtained, as quantified by densitometric comparison of the intensity of the detected nanobody band with a control sample of nanobody without nanogel or soluble polymer (Figure 3B).

We afterwards also checked whether nanobody conjugation would affect the integrity of the polymeric carrier and performed additional DLS experiments, as summarized by Table 1 and Figure 2A. Due to the small size of the nanobodies of only 15 kDa, they hardly affect the hydrodynamic diameter of the nanogels or polymers. Moreover, their conjugation does also not influence the pH-responsiveness of the ketal-crosslinked, degradable nanogel. Upon exposure to acidic pH-values, the nanobody-modified nanogel still falls apart into single polymer chains in analogy to the non-modified nanogel (Figure 2A).

Additionally, UV-vis spectroscopy measurements were performed to prove that the covalently attached fluorescent dye is not affected by the conjugation of the nanobody. Figure 2B shows that despite the different carrier species (nanogel or soluble polymer) with individual degradation profiles and nanobody surface modifications, their fluorescent dye labeling is still preserved by almost similar absorption maxima for the fluorescent dye Oregon Green around 490 nm. Consequently, its fluorescent signal can be used as readout for particle association and uptake in MMR expressing cells by both confocal microscopy or flow cytometry.

Next, cells were incubated with the nanogel species with or without nanobody modification in order to study a nanobody-mediated binding and internalization into the target cells. For that purpose, immortalized Chinese Hamster Ovary cells were used that were genetically modified to stably express the MMR/CD206 receptor. Both cell lines, either CHO^MMR−^ or CHO^MMR+^, were incubated at 50 µg/mL with the corresponding nanogel or polymer samples for 4 h at 37 °C and subsequently analyzed by flow cytometry.

**Figure 4:**
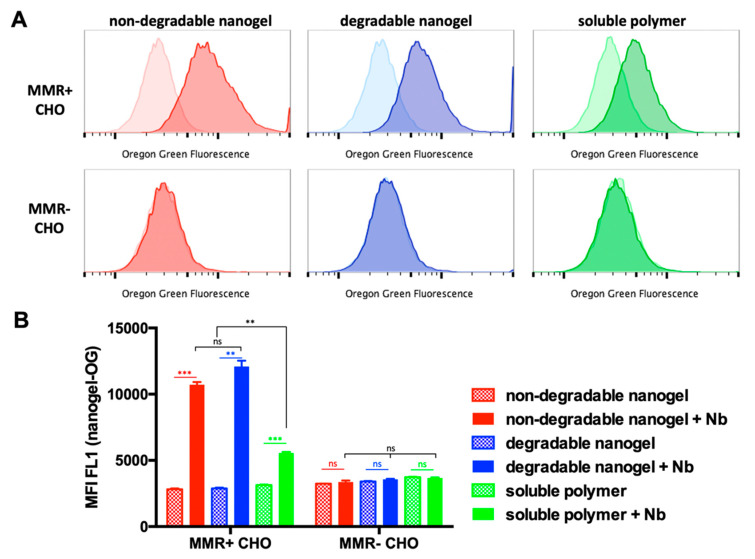
Flow cytometric analysis of CHO^MMR−^ or CHO^MMR+^ cells incubated with (non-)degradable nanogels or polymers, conjugated with or without MMR-targeting nanobody, at 50 µg/mL for 4 h at 37 °C. (**A**) Flow cytometric histograms and (**B**) corresponding mean fluorescence intensity (MFI) plots (*n* = 3). (**: *p* ≤ 0.001; ***: *p* ≤ 0.0001).

Flow cytometric histograms are shown in Figure 4, illustrating that only after nanobody conjugation a high amount of Oregon green positive cells could be found exclusively for the CHO^MMR+^ cells, indicating that this internalization is clearly mediated by the MMR-targeting nanobody (Figure 4A). In line with this conclusion, CHO^MMR−^ did not internalize any nanocarrier with or without nanobody. Interestingly, both non-degradable and degradable nanogel conjugated with MMR-targeting nanobodies provided similar, but much stronger Oregon Green fluorescence, compared to the soluble polymer-nanobody conjugate.

This could further be confirmed by comparing the cells’ mean fluorescent intensity (MFI) (Figure 4B). For both non-degradable and degradable nanogel conjugated with MMR-targeting nanobodies, identical MFI values could be measured that were significantly higher than for the soluble polymers with nanobodies on the CHO^MMR+^ cells. All other samples provided lower MFI values in analogy to all samples on the CHO^MMR−^ cells (Figure 4B). Consequently, these results confirm that nanobodies enable a specific targeting for the nanocarriers. Most interestingly, this precision is even enhanced, when the nanobody is presented in a multivalent way on the surface of an intact nanogel, probably because of the enhanced interaction capabilities of the carrier with the target receptor compared to a single polymer nanobody conjugate (compare Figure 6).

**Figure 5:**
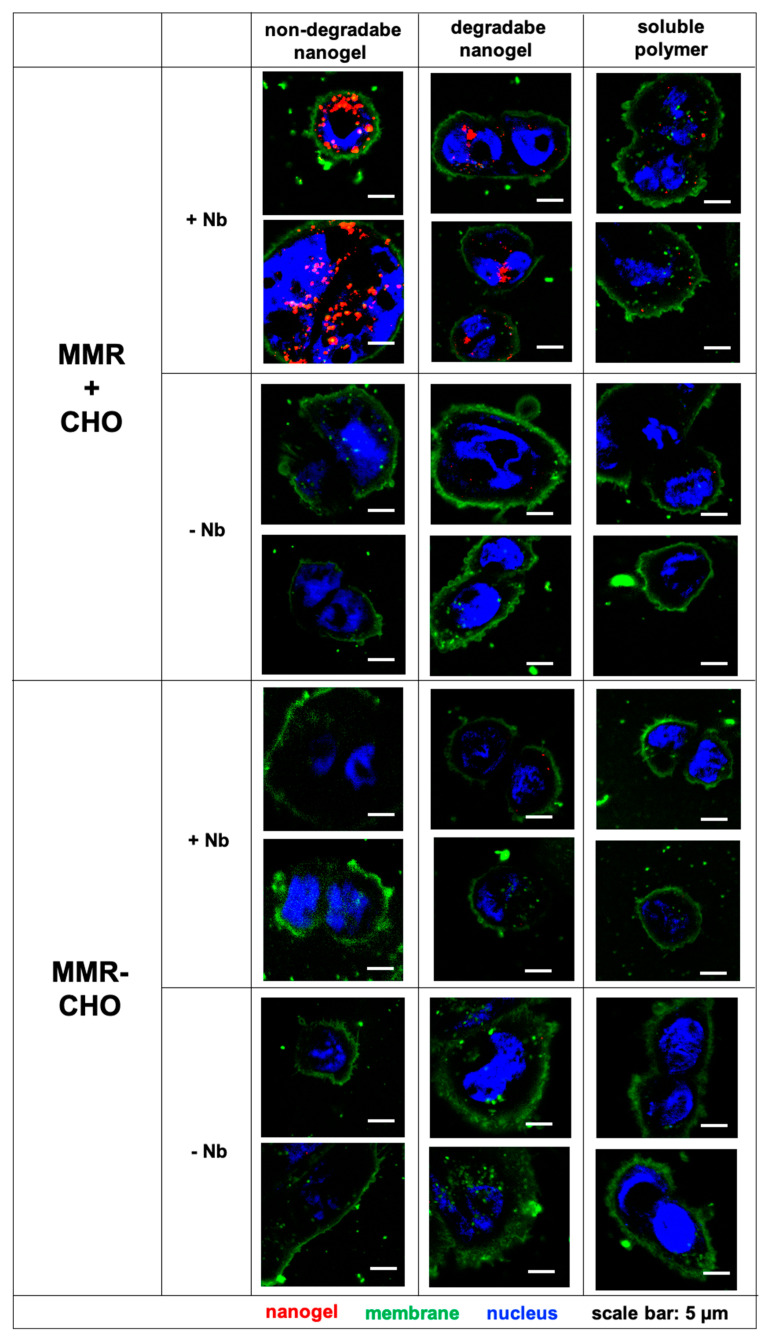
Fluorescence confocal images of CHO^MMR−^ or CHO^MMR+^ cells incubated with (non-)degradable nanogels or polymers, conjugated with or without MMR-targeting nanobody, at 50 µg/mL for 4 h at 37 °C. Nuclei are stained with DAPI (blue) and membranes were labeled with Alexa Fluor 555 phalloidin (green), while the (non-)degradable nanogels or polymer covalently modified with Oregon Green are depicted in red.

To corroborate these observations, we performed additional confocal microscopy experiments on both CHO^MMR−^ and CHO^MMR+^ cells to verify the internalization of the nanogels and polymers. As shown in Figure 5, exclusively CHO^MMR+^ cells showed nanogel-derived intracellular fluorescence after incubation with nanobody-modified nanogels or soluble polymers. All other samples did not show any carrier derived fluorescence inside the cell. Interestingly, in accordance with our flow cytometry data, the fluorescence of the two types of nanobody-modified nanogels inside the CHO^MMR+^ cells was again much more intense than for the soluble polymer-nanobody conjugates. No nanogel-derived fluorescence could be found for any sample in CHO^MMR−^ cells (Figure 5).

**Figure 6:**
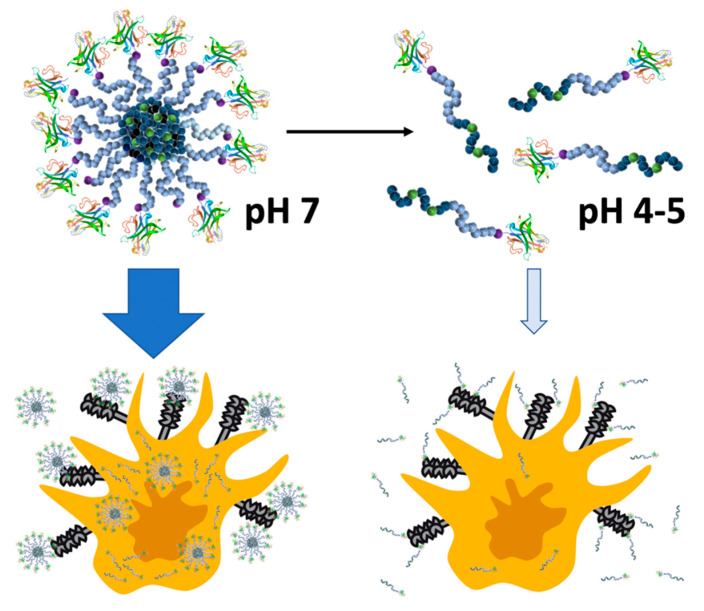
Transient multivalent nanobody targeting to MMR (CD206)-expressing cells via acidic pH-degradable nanogels. As intact particles, they display the conjugated nanobody on their surface in a multivalent way and, therefore, provide a higher affinity to MMR (CD206)-expressing cells. After endosomal acidification the nanogels unfold into single polymer chains with a reduced affinity to those cells.

Altogether, these additional results clearly confirm that the MMR-targeting nanobody is an excellent device to precisely target MMR expressing cells, both in vitro as well as in vivo, as demonstrated earlier [34]. Moreover, this targeting capacity is especially enhanced when the nanobody is presented in a multivalent way on a nanogel carrier surface compared to a single soluble polymer chain, independent from the nanogel degradation profile. The latter, however, is an attractive feature to control nanobody targeting. After internalization, the ketal-crosslinked carriers can fall apart into soluble polymers [45], thereby reducing their targeting capacity and, thus, providing a transient and controllable targeting feature for these types of nanogels. These conclusions are summarized by Figure 6.

## 4. Summary and Conclusions

Nanobodies are an attractive tool to target specific cells, especially in the immune system, as they lack the Fc part compared to traditional antibodies and, thus, prevent Fc-receptor mediated unfavorable mistargeting. We have applied nanobodies as targeting entities on three types of dye-labeled nanogel derivatives derived from the same reactive ester precursor block copolymer (non-degradable nanogels, acid-degradable nanogels (with ketal crosslinks), and single polymer chains (as their degraded version)). Via an SPAAC conjugation strategy, all of them could be conjugated to DBCO-modified anti-MMR nanobodies and subsequently incubated with naïve and MMR-expressing Chinese hamster ovary (CHO) cells. By both confocal microscopy and flow cytometry, a receptor-specific targeting and uptake could be confirmed for the nanocarriers. Interestingly, the intact nanogels displaying nanobodies in a multivalent fashion showed a much stronger binding and uptake than the soluble polymers as their degraded version. Due to the acidic pH-responsive degradation potential of the ketal-crosslinked nanogels, these carriers are able to facilitate a transient targeting that gets diminished upon unfolding into single polymer chains after endosomal acidification. Such control over particle integrity and targeting performance can be considered as highly suitable for safe and controllable immunodrug delivery purposes.

## Figures and Tables

**Table 1:** Characterization of non-degradable nanogels, degradable nanogels, and their corresponding soluble polymer chains with and without conjugated nanobodies.

Sample	Precursor Polymer	Crosslinker	Hydrodynamic Diameter [Nm] *	Poly-Dispersity *
non-degradable nanogel	N_3_-(mTEGMA)_12_ -*b*-P(PFPMA)_40_	2,2′ -(ethylenedioxy) bis(ethylamine)	51.2 ± 0.5	0.23 ± 0.01
non-degradable nanogel + nanobody	N_3_-(mTEGMA)_12_ -*b*-P(PFPMA)_40_	2,2′ -(ethylenedioxy) bis(ethylamine)	57.6 ± 0.6	0.33 ± 0.01
degradable nanogel	N_3_-(mTEGMA)_12_ -*b*-P(PFPMA)_40_	2,2-bis(amino-ethoxy)propane	61.9 ± 0.5	0.20 ± 0.01
degradable nanogel + nanobody	N_3_-(mTEGMA)_12_ -*b*-P(PFPMA)_40_	2,2-bis(amino-ethoxy)propane	58.3 ± 0.5	0.22 ± 0.01
soluble polymer	N_3_-(mTEGMA)_12_ -*b*-P(PFPMA)_40_	2-ethanolamine (non-crosslinking)	9.4 ± 0.6 ^#^	0.64 ± 0.26
soluble polymer + nanobody	N_3_-(mTEGMA)_12_ -*b*-P(PFPMA)_40_	2-ethanolamine (non-crosslinking)	8.9 ± 3.2 ^#^	0.57 ± 0.15

* Determined by dynamic light scattering (DLS) at 0.5 mg/mL in PBS. ^#^ Volume mean. All data are mean average of three measurements with standard deviation.
